# Autologous Fat Grafting (AFG): A Systematic Review to Evaluate Oncological Safety in Breast Cancer Patients

**DOI:** 10.3390/jcm13154369

**Published:** 2024-07-26

**Authors:** Federico Lo Torto, Luca Patanè, Donato Abbaticchio, Alessia Pagnotta, Diego Ribuffo

**Affiliations:** 1 Plastic Surgery Unit, Department of Surgery, Sapienza University of Rome, 00185 Rome, Italy; 2 Hand and Microsurgery Unit of the Jewish Hospital of Rome, 00186 Rome, Italy

**Keywords:** autologous fat grafting, breast cancer, recurrence, lipofilling, LRR, breast reconstruction

## Abstract

**Background:** Autologous fat grafting (AFG) has emerged as a useful technique in breast reconstruction. Utilizing a patient’s own fat from areas like the abdomen or thighs, AFG serves various reconstruction needs. Nevertheless, the oncological safety of AFG in breast cancer patients has become a contentious issue. Concerns about its influence on cancer recurrence and detention have led to significant clinical debate and the need for thorough investigation. **Methods:** To determine the impact of autologous fat grafting (AFG) on loco-regional recurrence (LRR) in breast cancer survivors undergoing reconstruction, a comprehensive search of databases including PubMed, Medline, Web of Science, and Cochrane libraries was conducted from November 2023 through March 2024. This search adhered to the PRISMA guidelines and aimed to identify all the relevant studies on AFG in the context of breast reconstruction post cancer treatment. A meta-analysis was performed. **Results:** Out of the studies reviewed, 40 met the inclusion criteria, with a total patient cohort of 14,078. The analysis revealed that AFG had no significant association with increased rates of LRR. **Conclusions:** According to the available literature, AFG is a safe reconstructive option for breast cancer patients and does not increase the risk of loco-regional recurrence. Nevertheless, further well-structured long-term prospective studies are required, since heterogeneity of available studies is high and requires standardization.

## 1. Introduction

Breast cancer constitutes a prominent global health issue, impacting a significant number of women worldwide and presenting complex challenges for both patients and healthcare professionals. It ranks as the most commonly diagnosed cancer in a majority of nations (154 out of 185) and is the foremost cause of cancer-related deaths in over 100 countries [1]. The incidence of local–regional recurrences (LRR) following breast cancer surgery plays a critical role in mortality and disease-free survival (DFS), which, in turn, serves as a reliable surrogate marker for overall survival [2].

Over recent decades, the surgical management of breast cancer has shifted from more radical procedures to those conserving breast tissue. Efforts by oncologists and plastic surgeons are increasingly focused not only on enhancing oncological treatments but also on advancing reconstructive techniques to address contour defects and restore volume, aiming to improve outcomes and patient quality of life [3,4]. The necessity for demolitive surgical approaches, such as mastectomy or breast-conserving surgery (BCS), can significantly alter a patient’s physical appearance and self-perception, thereby impacting their quality of life [5]. This alteration often leads individuals to pursue reconstructive surgery as a means to reclaim their sense of femininity and integrity post treatment.

In recent years, autologous fat grafting (AFG), has gained increasing attention as a valuable adjunctive technique in breast reconstruction following demolitive surgery. This approach involves the transplantation of a patient’s own adipose tissue harvested from donor sites, such as the abdomen or thighs, to address different necessities of the patient. AFG has several indications in breast reconstructive surgery as an ancillary procedure to address asymmetry corrections following BCS [6], contour irregularities of the reconstructed breast [7], thinning of the subcutaneous tissue to prevent expander/implant exposure before [8] or after RT [9,10], and as the sole procedure for reconstruction of a small-sized breast [11]. For the latter, AFG offers several advantages over traditional implant-based or flap reconstruction methods, including its ability to achieve more natural-looking results, enhance breast symmetry, have low incidence of revision surgeries and minimize donor-site morbidity. Nevertheless, oil cysts, fat necrosis and macrocalcifications may occur, mainly following high-volume transfers [12].

Despite its growing popularity and perceived benefits, concerns regarding the oncologic safety of AFG in breast cancer patients have emerged as a topic of considerable debate and scrutiny within the medical community [13,14,15]. Questions regarding its potential impact on cancer detection [16,17], promotion of tumor growth [18], and facilitation of local recurrence [19,20,21,22] have raised important clinical considerations and prompted calls for rigorous evaluation.

The existing literature on the oncologic safety of breast AFG is characterized by a heterogeneous array of studies with varying methodologies, patient populations, and outcomes, resulting in conflicting findings and inconclusive evidence. The recent increase in research on this topic calls for an updated analysis that combines the latest studies with past evidence. By synthesizing and critically appraising the existing literature, this review aims to provide a comprehensive overview of the safety profile of breast AFG in the context of oncologic surgery.

## 2. Materials and Methods

We performed a systematic review of the literature in accordance with PRISMA guidelines, and was not registered in any systematic review registry. We searched for publications on PubMed, Embase, Web of Science (including Science Citation Index and Conference Proceedings Citation Index), and Cochrane Library databases to identify all publications regarding autologous fat grafting after breast cancer surgery. For all the libraries, the following search term strategy was used: (autologous fat grafting [MeSH] AND breast [MeSH]). As there are several different terms describing autologous fat grafting, and to maintain a systematic approach, available synonyms were also used as search terms. The used Mesh-terms were the following: autologous fat transfer, lipofilling, adipose fat transfer, lipotransfer, adipose tissue, breast cancer, fat grafting, and cancer recurrence. All citations were screened through their titles and abstracts, duplicates were removed, and then full-text manuscripts were assessed according to the following inclusion criteria: only human-based topics and manuscripts written in English were to be taken into consideration. Case reports and case series with less than 15 patients, letters, review, book chapters, or a Jadad modified scale score <2 were used as exclusion criteria for this review.

The review search started on November 2023 and ended in March 2024 and was conducted by L.P. and D.A. The two reviewers independently reviewed the titles and abstracts yielded by this comprehensive search and subsequently selected articles based on the predetermined inclusion and exclusion criteria. Disagreements were resolved through consensus-based discussion with a third reviewer (F.L.T.).

The following data were extracted from the manuscripts included in the final tally: study period, number of patients, mean age, type of surgery before autologous fat grafting, incidence of invasive carcinomas, carcinoma in situ (CIS), radiotherapy (RT) before autologous fat grafting, mean time between surgery and autologous fat grafting, mean follow-up period after autologous fat grafting, and number of patients with local recurrence. The endpoints of this study were to analyze the correlation between AFG and LRR rates and to analyze the factors implicated in a higher incidence of LRR, such as percentage of invasive carcinoma, percentage of RT and follow-up. The level of evidence for included studies was evaluated using the Oxford Centre for Evidence-Based Medicine (OCEBM) [23] and the Oxford quality scoring system (Jadad Score) [24], which are instruments to evaluate the quality of observational and randomized studies.

The meta-analysis was performed using the MetaXL 5.3 software, and the meta-regression was performed using SPSS Statistics v28.0 (IBM, Armonk, NY, USA). The meta-analysis was conducted in distinct phases. First, we assessed the LRR in studies that compared groups of patients undergoing AFG with those who did not receive AFG. Following this comparative analysis, the second phase examined LRR in single-arm studies, comparing with the overall LRR prevalence. Lastly, separate meta-analyses were performed comprising only matched and unmatched studies. A meta-regression analysis was subsequently performed to investigate the impact of the percentage of invasive carcinomas, the proportion of patients receiving radiotherapy, and the follow-up duration on LRR rates.

## 3. Results

### 3.1. Study Selection

**Figure 1:**
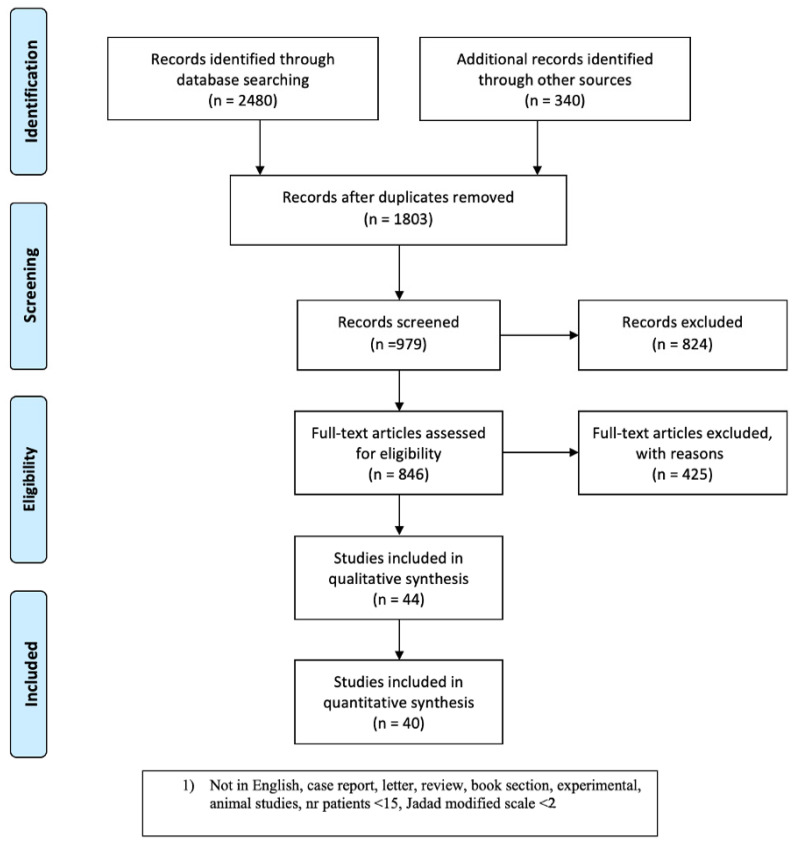
Flow diagram representation of the search strategy used for the systematic review, in accordance with PRISMA guidelines.

From 2480 starting citations scrutinized in the study period, we identified 1803 articles following the first screening based on the assessment of titles and abstracts. Any citation deemed not relevant to the systematic review endpoints was excluded. After duplicates were excluded, 979 articles were screened and manuscripts not meeting the inclusion criteria or meeting the exclusion criteria were discarded, only leaving 846 articles. After full-text assessment, any manuscript that did not provide clinical data of a patient population undergoing AFG following breast cancer was excluded. Data from 40 manuscripts were included for analysis. A flow chart representation of the search strategy with the included and excluded articles is depicted in Figure 1.

### 3.2. Analysis of Selected Studies

Seven studies [25,26,27,28,29,30,31] reported a higher rate of LRR in the population of patients who underwent AFG. Seven studies [32,33,34,35,36,37,38] reported a comparable rate of LRR between patients who underwent AFG and patients that did not undergo AFG. Seven studies [39,40,41,42,43,44,45] reported a lower rate of LRR in the population of patients who underwent AFG. Nineteen studies [46,47,48,49,50,51,52,53,54,55,56,57,58,59,60,61,62,63,64] reported LRR rates only for patients who underwent AFG.

### 3.3. Comparative Analysis

Shown in Table 1 is an outline of clinical investigations regarding the potential oncological hazards associated with surgical procedures for breast cancer, related to LRR rate. Among 6459 patients who received mastectomy or breast conservative surgery (MST/BCS) without incorporating AFG, the analysis demonstrated an LRR of 5.3%. This indicates that 342 patients encountered local recurrence. A total of 7619 patients underwent AFG following MST or BCS. Of these, 240 patients experienced a loco-regional recurrence, accounting for 3.15%.

### 3.4. Meta-Analysis

**Figure 2:**
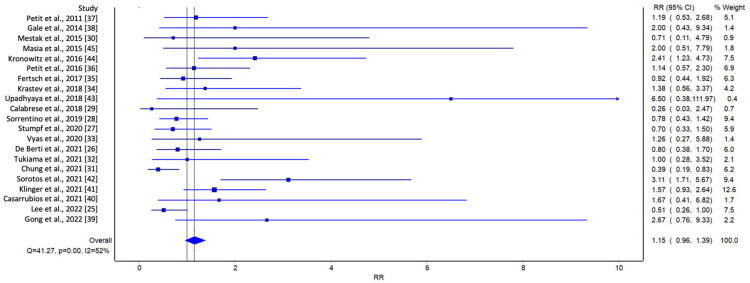
Meta-analysis evaluating LRR in comparative studies. Overall prevalence of LRR was used to compare each study [25,26,27,28,29,30,31,32,33,34,35,36,37,38,39,40,41,42,43,44,45].

**Figure 3:**
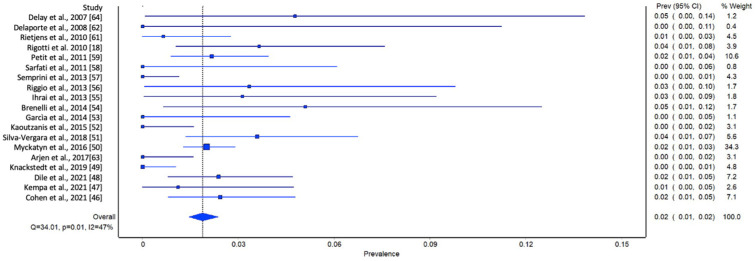
Meta-analysis evaluating the prevalence of LRR in single-arm studies. Overall prevalence of LRR was used to compare each study [18,46,47,48,49,50,51,52,53,54,55,56,57,58,59,61,62,63,64].

In the initial analysis of comparative studies (Figure 2), a high degree of study heterogeneity was noted. No direct correlation between AFG and LRR was identified. However, even though it was not statistically significant, there was an observable trend favoring AFG. This trend was further corroborated with the cumulative prevalence analysis (Figure 3).

It was not possible to conduct a comprehensive meta-analysis that examined all the various subgroups extracted from the studies, such as histology, receptor status, timing of AFG, and type of surgery. This was due to the fragmented nature of the data reported and the inconsistent presence of these variables across all the studies examined. Consequently, a meta-regression was performed, identifying two major groups: comparative studies [25,26,27,28,29,30,31,32,33,34,35,36,37,38,39,40,41,42,43,44,45] and single-arm studies [46,47,48,49,50,51,52,53,54,55,56,57,58,59,60,61,62,63,64].

**Figure 4:**
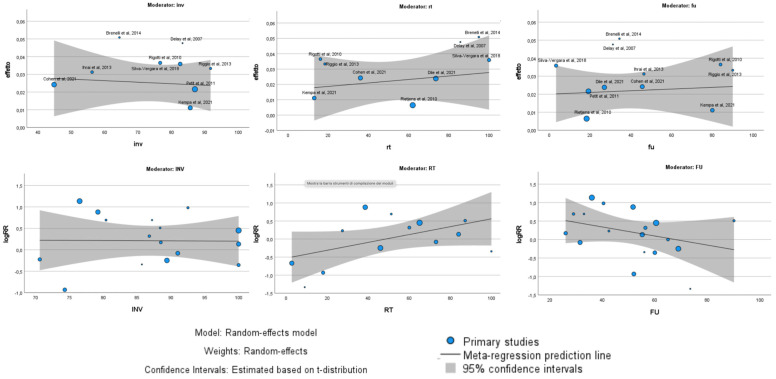
Meta-regression analysis with Bubble Plot of single-arm studies (**above**) and comparative studies (**below**). Correlations with percentage of invasive carcinomas, percentage of radiotherapy, and follow-up were analyzed.

Within these groups, the oncological outcome, specifically LRR, was analyzed by examining possible subgroups, including the percentage of invasive carcinomas, the percentage of patients undergoing radiotherapy, and the follow-up duration (Figure 4). The meta-regression analysis revealed distinct findings between single-arm and comparative studies regarding the factors influencing the oncological outcomes of autologous fat grafting (AFG). In single-arm studies, no significant relationship was found between the outcomes and the percentage of invasive carcinomas (*p* = 0.74), the percentage of patients receiving radiotherapy (*p* = 0.54), or the duration of follow-up (*p* = 0.77). This suggests that in isolated evaluations of AFG, these variables did not substantially impact the effectiveness or results of the procedure. In contrast, comparative studies presented a more nuanced picture. The percentage of invasive cancers remained non-influential on the results (*p* = 0.79), maintaining consistency with the single-arm studies. However, a trend emerged indicating a potential disadvantage for AFG with longer follow-up durations, although this trend was not statistically significant (*p* = 0.06). Moreover, a statistically significant relationship was observed with the percentage of patients receiving radiotherapy. Specifically, as the percentage of radiotherapy-treated patients increased, the outcomes for AFG improved significantly (*p* = 0.009).

**Figure 5:**
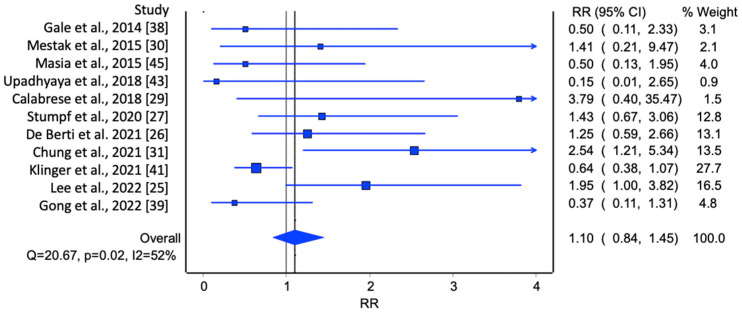
Meta-analysis evaluating the prevalence of LRR in unmatched studies [25,26,27,29,30,31,38,39,41,43,45].

**Figure 6:**
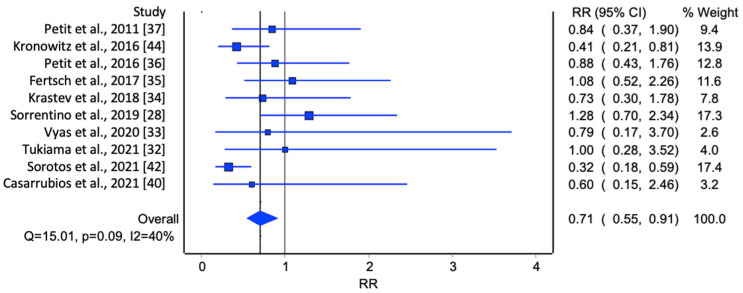
Meta-analysis evaluating the prevalence of LRR in matched studies [28,32,33,34,35,36,37,40,42,44].

Lastly, two meta-analyses were conducted, one including only unmatched studies (Figure 5) and the other including only matched studies (Figure 6). In the first group, the meta-analysis included unmatched studies, which may introduce more variability and potential confounding factors, whereas the studies included in the second meta-analysis were conducted with patient matching, which means the patient groups were more comparable, potentially reducing bias. For the first meta-analysis, the overall RR was 1.10 (95% CI: 0.84, 1.45). Even though there was an increase in the risk of loco-regional recurrence for patients who underwent breast lipofilling compared to those who did not, this result was not statistically significant. The heterogeneity statistics indicated moderate to substantial variability among the study results, suggesting that the results from these studies are not highly consistent. For the second meta-analysis, the overall RR was 0.71 (95% CI: 0.55, 0.91). This result suggests a 29% reduction in the risk of loco-regional recurrence for patients who underwent breast lipofilling compared to those who did not, and this reduction was statistically significant. The heterogeneity statistics indicated moderate variability among the study results, suggesting that while the studies were not perfectly consistent, they were reasonably comparable.

**Figure 7:**
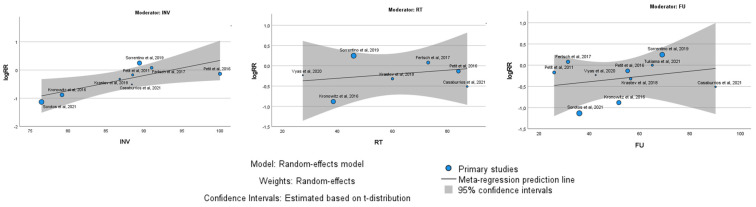
Meta-regression analysis with Bubble Plot of studies with patients matching. Correlations with percentage of invasive carcinomas, percentage of radiotherapy, and follow-up were analyzed.

The meta-regression analysis (Figure 7) showed a slight positive association with percentage of invasive carcinomas (*p* = 0.03).

**Table 1:** Literature review.

Author	Year of Publication	Type of Study	Group	Study Period	Patients (n)	Mean Age (y)	Type of Surgery	Invasive Carcinomas (n)	In Situ Carcinomas (n)	RT before AFG (%)	Mean Surgery-AFG (m)	Mean Follow-up (m)	LRR (%)	LRR (n)
Delay et al. [64]	2007	Observational study	BCS + AFG	2002–2007	42	51	BCS + AFG	35	3	85.70	78	31.2	4.76	2
Delaporte et al. [62]	2008	Observational study	MST + AFG	2002–2007	15	50	MST + AFG	9	6	78.50	N/A	27.6	0	0
Rietjens et al. [61]	2010	Observational study	BCS + MST + AFG	2005–2008	155	48	BCS + MST + IBR + ABR + AFG	N/A	N/A	62	50.5	18.3	0.70	1
Rigotti et al. [18]	2010	Retrospective cohort study	MST +AFG	2000–2005	137	46.5	MST +AFG	105	31	16.10	3.2	84	3.65	5
Petit et al. [37]	2011	Multicenter retrospective study	BCS + MST + AFG	2000–2010	370	52	BCS + MST +AFG	87	13	N/A	N/A	19.2	2.16	8
Petit et al. [59]	2011	Matched cohort study	MST	1997–2008	642	46	MST	568	74	N/A	N/A	26	3	19
			MST + AFG		321	45	MST + AFG	284	37	N/A	26	26	2.50	8
Sarfati et al. [58]	2011	Prospective study	MST + AFG	2007–2009	28	45	MST + AFG	N/A	N/A	100	N/A	17	0	0
Semprini et al. [57]	2013	Observational study	BCS + AFG	2006–2012	151	N/A	BCS + AFG	N/A	N/A	N/A	24	45	0	0
Riggio et al. [56]	2013	Observational study	MST + AFG	2000–2007	60	49.7	MST + AFG	55	5	18.30	55.2	90	3.30	2
Ihrai et al. [55]	2013	Retrospective study	MST + AFG	2004–2009	64	N/A	MST + AFG	36	10	N/A	N/A	46.44	3.10	2
Brenelli et al. [54]	2014	Prospective study	BCS + AFG	2005–2008	59	50	BCS + AFG	38	7	94.90	N/A	34.4	5.10	3
Gale et al. [38]	2014	Clinical study	BCS + MST	2007–2013	422	48.2	BCS + MST	368	54	N/A	54	34	1.90	8
			BCS + MST + AFG		211	47	BCS + MST + AFG	184	27	108	54	32	0.95	2
Garcìa et al. [53]	2014	Observational study	BCS + AFG	N/A	37	55	BCS + AFG	0	37	N/A	0	1	0	0
Kaoutzanis et al. [52]	2015	Retrospective study	MST + AFG	2008–2013	108	48	MST + AFG	68	40	23.30	10.8	20.2	0	0
Mestak et al. [30]	2015	Prospective study	BCS	2011–2014	45	64	BCS	41	3	N/A	N/A	56	4.88	2
			BCS + AFG		32	53	BCS + AFG	25	4	100	77	56	6.25	2
Silva-Vergara et al. [51]	2015	Retrospective study	BCS + MST + AFG	2007–2015	195	52	BCS + MST + AFG	161	44	100	4	3.3	3.58	7
Masia et al. [45]	2015	Retrospective study case-control	MST + ABR	1989–2017	107	49	MST + ABR	87	16	N/A	N/A	29	5.60	6
			MST + ABR + AFG		107	49.19	MST + ABR + AFG	85	14	N/A	N/A	29	2.80	3
Kronowitz et al. [44]	2016	Retrospective cohort study	MST	2001–2014	670	46.5	MST	548	61	N/A	N/A	43.8	4.10	27
			BCS + MST + AFG		719	47.1	BCS + MST + AFG	552	108	38.50	2.63	59.6	1.60	12
Myckatyn et al. [50]	2016	Multicenter case cohort study	MST + AFG	2006–2011	1197	47	MST + IBR + ABR	N/A	N/A	N/A	N/A	N/A	11.00%	24
Petit et al. [36]	2016	Matched case-control study	MST	2006–2013	322	N/A	MST + BCS	322	0	86	N/A	52.8	5	16
			MST + AFG		322	N/A	MST + BCS + AFG	322	0	84	N/A	57.6	4.30	14
Arjen et al. [63]	2017	Retrospective cohort study	BCS + AFG	2008–2016	109	55	BCS + AFG	N/A	N/A	100	18	26.4	0	0
Fertsch et al. [35]	2017	Matched retrospective cohort study	MST + DIEP	2009–2013	100	50.7	MST + ABR	91	9	N/A	N/A	31	12	12
			MST + DIEP + AFG		100	49.6	MST + ABR + AFG	91	9	73	40.5	32	13	13
Krastev et al. [34]	2018	Matched cohort study	MST + BCS	2006–2014	300	49.4	MST + BCS	260	40	N/A	N/A	52.8	3.60	11
			MST + BCS + AFG		300	48.1	MST + BCS + AFG	261	39	60	N/A	60	2.60	8
Upadhyaya et al. [43]	2018	Retrospective chart review study	MST	2011–2016	449	N/A	MST + IBR + ABR	N/A	N/A	N/A	N/A	26	1.70	8
			MST + AFG		171	50.51	MST + IBR + ABR + AFG	N/A	N/A	N/A	N/A	26	0	0
Calabrese et al. [29]	2018	Prospective multi arm single center cohort study	MST	2008–2011	72	47.7	MST	N/A	N/A	N/A	N/A	72	1.60	1
			MST + AFG		57	50.3	MST + AFG	N/A	N/A	9	9	75	4.70	3
			MST + EAFG		54	48.8	MST +EAFG	N/A	N/A	17	10	84	2.40	1
Sorrentino et al. [28]	2019	Retrospective exact matching study	MST + BCS	2007–2017	597	50.7	MST + BCS	535	62	N/A	N/A	63.8	5.00%	30
			MST + BCS + AFG		233	49.4	MST + BCS + AFG	207	26	45.90	22.9	74.1	6.40	15
Knackstedt et al. [49]	2019	Retrospective cohort study	MST + IBR + AFG	2006–2015	166	52	MST + IBR + AFG	106	52	20	N/A	28	0	0
Stumpf et al. [27]	2020	Matched retrospective cohort study	BCS	2004–2016	255	54	BCS	255	0	N/A	N/A	60	8.60	22
			BCS + AFG		65	53	BCS + AFG	65	0	N/A	N/A	60	12.30	8
Vyas et al. [33]	2020	Matched case-control study	MST	2000–2017	69	N/A	MST	N/A	N/A	N/A	N/A	42.5	8.50	6
			MST +AFG		29	48.6	MST + AFG	N/A	N/A	27.4	N/A	42.5	8.20	2
Dile et al. [48]	2021	Retrospective study	MST + BCS + AFG	2013–2016	252	50	MST + BCS + ABR + IBR + AFG	N/A	N/A	73.50	35	27	2.40	6
Kempa et al. [47]	2021	Monocentric cohort study	MST + BCS + AFG	2008–2020	90	46.1	MST + BCS + AFG	77	13	13	57	80	0.90	1
De Berti et al. [26]	2021	Retrospective monocentric case-control study	MST	2007–2017	303	52	MST + BCS + ABR + IBR	202	87	N/A	N/A	N/A	6.60	20
			MST + AFG		109	50	MST + BCS + ABR +IBR + AFG	89	16	N/A	N/A	N/A	8.30	9
Tukiama et al. [32]	2021	Retrospective matched cohort study	MST	2007–2016	126	N/A	MST + BCS	N/A	N/A	N/A	N/A	65	7.10	9
			MST + AFG		42	N/A	MST + BCS + AFG	N/A	N/A	N/A	N/A	65	6.30%	3
Chung et al. [31]	2021	Retrospective cohort study	MST	2009–2019	272	50.4	MST + BCS + ABR + IBR	200	66	N/A	N/A	52	6	16
			MST + AFG		67	50.4	MST + BCS + ABR + IBR + AFG	52	15	18	N/A	52	15	10
Sorotos et al. [42]	2021	Retrospective matched case control study	MST	2005–2017	494	45 -49	MST + IBR + ABR	379	115	N/A	N/A	36	9.60	47
			MST + AFG		425	45–49	MST + IBR + ABR + AFG	324	101	N/A	N/A	36	3	13
Klinger et al. [41]	2021	Retrospective multicenter study case- control	MST + BCS	2000–2018	923	52.9	MST + BCS	923	N/A	N/A	N/A	58	6.10	56
			MST +BCS + AFG		466	51.4	MST + BCS + AFG	466	N/A	65	N/A	63	3.90	18
Casarrubios et al. [40]	2021	Matched cohort study	MST	2011–2019	125	47.2	MST + BCS	115	10	N/A	N/A	85	4	5
			MST + AFG		125	45.6	MST + BCS + AFG	106	19	87.20	48.1	95.3	2.40	3
Cohen et al. [46]	2021	Retrospective cohort study	MST + AFG	2010–2015	248	47.95	MST + AFG	111	51	36	13.2	45.6	2.40	6
Lee et al. [25]	2022	Retrospective cohort study	MST	2011–2016	126	43.9	MST + IBR	N/A	N/A	N/A	N/A	N/A	9	11
			MST + AFG		141	43.9	MST + IBR + AFG	N/A	N/A	2.70	12	N/A	17	24
Gong et al. [39]	2022	Retrospective cohort study	BCS	2018	40	50.8	BCS	36	4	N/A	N/A	40.28	10	8
			BCS + AFG		40	50.2	BCS + AFG	38	2	N/A	N/A	40.58	7.50	3

MST: Mastectomy; BCS: Breast conserving surgery; AFG: Autologous fat grating; EAFG: Enriched autologous fat grafting; IBR: Implant-based reconstruction; ABR: Autologous breast reconstruction.

## 4. Discussion

The oncological safety of AFG in breast reconstruction has been a subject of considerable debate for decades, stemming from diverse findings in cellular, biological, and clinical studies. This debate centers on whether the clinical advantages of AFG surpass its possible hazards. In our review we analyzed a total of 7619 patients who underwent AFG, with a total incidence of LRR of 3.15% and a total of 6459 patients who did not undergo AFG, with an LRR rate of 5.3% (Table 2).

A total of 40 articles were included in the evaluation and a meta-analysis was performed, highlighting the importance of this study to possibly overcome previous attempts to evaluate the oncological safety of AFG. In fact, previous reviews were hindered by the small number of articles reviewed.

From our analysis, even though the heterogeneity of the studies was wide, no direct correlation could be found between AFG and an increased risk of LRR.

Although conducting a comprehensive meta-analysis that included all subgroups from the studies was not feasible, a meta-regression analysis was carried out focusing on single-arm studies and comparative studies.

For single-arm studies, factors such as the percentage of invasive carcinomas, percentage of patients receiving radiotherapy, or the duration of follow-up did not influence LRR rates. However, a trend indicating a potential disadvantage for AFG with longer follow-up periods was observed in comparative studies. This trend was not statistically significant. Additionally, a significant statistical relationship was found between the percentage of patients receiving radiotherapy and LRR rates. Specifically, as the percentage of radiotherapy-treated patients increased, the LRR rate for patients who underwent AFG was lower. Lastly, while the meta-analysis results for unmatched studies were statistically non-significant, the one performed with matched studies revealed a reduced LRR rate in the AFG group. This reduction was slightly inferior when considering studies with a higher percentage of invasive carcinoma.

Concerns have been raised regarding the potential oncological risks associated with the use of adipose-derived mesenchymal stem cells (ADMSCs) in autologous fat grafting (AFG), particularly their secreting factors that may interact with primary breast cancer cells [65,66]. It is postulated that these factors may help develop and maintain an inflammatory state, in which tissue regeneration is stimulated, but that, on the other hand, they contribute to the process of tumor genesis and progression [67,68,69,70]. For this reason, concerns about the placement of regenerative tissue in a tumor bed raised doubts about the oncological safety of AFG in this context. Even though the American Society of Plastic Surgeons set up a task force to assess the indications, safety, and efficacy of AFG [71], a low grade of scientific evidence was present, thus failing to provide specific recommendations on the topic.

Nevertheless, after almost 15 years, there is still not scientific evidence to support such a possibility.

Analyzing the results of our study, we believe that the heterogeneity of the included studies, variations in study methodologies, and a paucity of long-term follow-up data are the main issues that should be resolved to obtain high-quality studies. Additionally, the lack of standardized reporting and inconsistent definitions of outcomes across studies pose challenges in synthesizing and interpreting the findings. Some studies indicate that AFG has little to no effect on local recurrence or cancer progression, while other studies suggest a possible risk of tumor recurrence and complications in monitoring for cancer. In our study, conflicting results in terms of the LRR rate were observed. Therefore, in our opinion, the decision to incorporate breast AFG into clinical practice should be made judiciously, considering patient-specific factors, tumor characteristics, and potential oncologic risks. The lack of a clear correlation between AFG and LRR seems to underline the importance of other factors, such as oncological and surgical variables, in breast cancer recurrence.

## 5. Future Directions

Future research efforts should focus on prospective, multicenter studies with standardized protocols to elucidate the long-term oncologic outcomes of breast AFG and identify patient subgroups that may benefit most from this procedure. We believe that characteristics of the tumor (such as clinical stage, histology, etc.) and genetic factors may play an important role in LRR and must be analyzed in these types of studies. Overall survival (OS) and disease-free survival (DFS) are additional sources of information that should be included. Furthermore, investigations into the underlying mechanisms of tumor interactions with adipose-derived stem cells and the tumor microenvironment are warranted to understand the oncologic implications of breast AFG better and inform evidence-based clinical practice guidelines.

## 6. Conclusions

Despite the fact that the cautions of the American Society of Plastic Surgeons remain pertinent, the current literature supports that AFG is an oncologically safe procedure, whose routine use appears to be justified. The discrepancy between experimental and in vivo studies may be due to the complexity of oncological processes and the inability to recreate, in vitro, the intricacy of the in vivo microenvironment. Further well-structured long-term prospective studies are required for more solid evidence.

## Figures and Tables

**Table 2:** Risk of LRR with or without AFG.

	Patients (No.)	LRR (No)	LRR (%)
MST/BCS + AFG	7619	240	3.15%
MST/BCS	6459	342	5.30%

MST: Mastectomy; BCS: Breast conserving surgery; AFG: Autologous fat grating.

## Data Availability

Not applicable.
